# Use of procalcitonin for identification of postoperative complications after coronary artery bypass surgery with cardiopulmonary bypass

**DOI:** 10.1186/cc13412

**Published:** 2014-03-17

**Authors:** A Baysal, M Doğukan, H Torman

**Affiliations:** 1 Kartal Kosuyolu High Speciality Research and Training Hospital, Istanbul, Turkey; 2 Adtyaman University Training and Research Hospital, Adiyaman, Turkey; 3 Çanakkale 18 Mart Research Hospital, Çanakkale, Turkey

## Introduction

The values of C-reactive protein (CRP) and procalcitonin (PCT) were investigated to determine their effects on postoperative complications in patients with or without systemic inflammatory response syndrome (SIRS).

## Methods

In 183 patients, in a prospective observational study, serum CRP and PCT values were collected every day starting on postoperative day 1 through day 5. The definition of SIRS includes two or more of the following: temperature >38 or <36°C; heart rate >90 beats/minute; respiratory rate >20/minute; arterial carbon dioxide pressure <32 mmHg; white blood cell count >12,000/mm^3 ^and <4.00/mm^3^. The ability of PCT to predict sepsis and other postoperative complications were determined by performing receiver operative characteristic curve analysis.

## Results

All patients were divided post hoc into patients with SIRS (Group 1, *n *= 83) and patients without SIRS (Group 2, *n *= 100). A PCT threshold value of 2.79 ng/ml on postoperative day 1 was able to discriminate postoperative complications in patients with or without SIRS with a sensitivity of 82.5% and a specificity of 70% (area under curve: 0.76, *P *< 0.01).

## Conclusion

(1) Serum PCT values increased significantly after cardiopulmonary bypass in the SIRS group in comparison with patients without SIRS on postoperative day 1 and remain elevated until postoperative day 5. (2) Serum CRP values also follow a similar pattern, buta CRP threshold value was not obtained to differentiate between postoperative complications in patients with or without SIRS. A PCT threshold value of 2.79 ng/ml on postoperative day 1 is a valuable marker to discriminate between patients with or without SIRS.

